# Thiol-Responsive Gold Nanodot Swarm with Glycol Chitosan for Photothermal Cancer Therapy

**DOI:** 10.3390/molecules26195980

**Published:** 2021-10-02

**Authors:** SeongHoon Jo, In-Cheol Sun, Wan Su Yun, Jinseong Kim, Dong-Kwon Lim, Cheol-Hee Ahn, Kwangmeyung Kim

**Affiliations:** 1 Center for Theragnosis, Biomedical Research Institute, Korea Institute of Science and Technology, 5, Hwarang-ro, Seongbuk-gu, Seoul 02792, Korea; jsh@kist.re.kr (S.J.); pfesun@kist.re.kr (I.-C.S.); 2 Department of Materials Science and Engineering, Research Institute of Advanced Materials (RIAM), Seoul National University, 1 Gwanak-ro, Gwanak-gu, Seoul 08826, Korea; 3 KU-KIST Graduate School of Converging Science and Technology, Korea University, 145 Anam-ro, Seongbuk-gu, Seoul 02841, Korea; ip9801@kist.re.kr (W.S.Y.); 218843@kist.re.kr (J.K.); dklim@korea.ac.kr (D.-K.L.)

**Keywords:** gold nanoparticle, glycol chitosan, photothermal therapy, immunogenic cell death, thiol-responsiveness

## Abstract

Photothermal therapy (PTT) is one of the most promising cancer treatment methods because hyperthermal effects and immunogenic cell death via PTT are destructive to cancer. However, PTT requires photoabsorbers that absorb near-infrared (NIR) light with deeper penetration depth in the body and effectively convert light into heat. Gold nanoparticles have various unique properties which are suitable for photoabsorbers, e.g., controllable optical properties and easy surface modification. We developed gold nanodot swarms (AuNSw) by creating small gold nanoparticles (sGNPs) in the presence of hydrophobically-modified glycol chitosan. The sGNPs assembled with each other through their interaction with amine groups of glycol chitosan. AuNSw absorbed 808-nm laser and increased temperature to 55 °C. In contrast, AuNSw lost its particle structure upon exposure to thiolated molecules and did not convert NIR light into heat. In vitro studies demonstrated the photothermal effect and immunogenic cell death after PTT with AuNSW. After intratumoral injection of AuNSw with laser irradiation, tumor growth of xenograft mouse models was depressed. We found hyperthermal damage and immunogenic cell death in tumor tissues through histological and biochemical analyses. Thiol-responsive AuNSw showed feasibility for PTT, with advanced functionality in the tumor microenvironment.

## 1. Introduction

Cancer is one of the top 10 causes of death [1] that takes away everyday lives. To regain lives from cancer, researchers have contrived several novel therapeutic methods, such as targeted therapy, personalized medicine, and immunotherapy. Among these, immunotherapy is a cancer treatment method with numerous advantages. For example, immunotherapy is effective against various types of cancer because it enables our immune system to recognize cancer cells and attack them [2]. The active immune cells and their immunological memory against primary tumors prevent metastasis and cancer recurrence [3,4].

Recently, photothermal therapy (PTT) has attracted attention as an approach to immunotherapy. Photoabsorbers that convert light energy into heat upon light irradiation play a critical role in PTT, as hyperthermia leads to therapeutic effects against cancer cells through cell membrane disruption, protein denaturation, and DNA damage [5]. Additionally, hyperthermia causes dying cells to release danger-associated molecular patterns (DAMPs) that recruit various immune cells. For example, dendritic cells activate T cells, which kill cancer cells and reduce tumor volume [6,7]; tumor-specific memory T cells inhibit cancer recurrence [8]. This type of cell death via the activated adaptive immune response is called immunogenic cell death (ICD). Therefore, PTT is a promising methodology in cancer immunotherapy because the photothermal effect and systemic immune responses simultaneously remove the primary tumor and eliminate metastatic tumors.

However, cancer treatment with PTT but without exogenous photoabsorbers is often inefficient. Because most endogenous photoabsorbers are ubiquitous in the body regardless of cancer, delivery of light energy into deep tissue has been an obstacle to PTT. Light in the near-infrared (NIR) range and exogenous photoabsorbers with NIR light absorption are necessary in order to provide the effective hyperthermic effect of PTT. In particular, nanoparticle-based photoabsorbers have such desirable properties as NIR light absorption and photostability. Therefore, several nanoparticles such as gold, iron oxide, melanin, and Prussian blue that have shown excellent light conversion ability into heat have been tested as PTT agents [7,9,10,11].

**Scheme 1:**
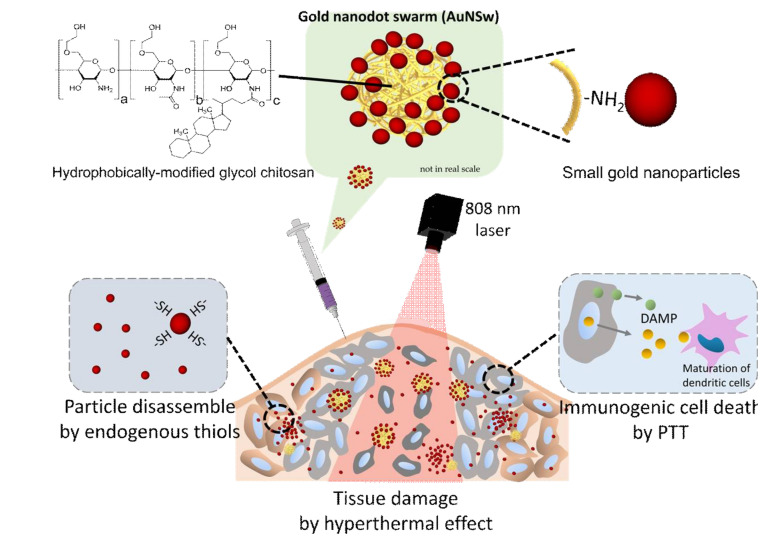
A schematic diagram of the particle structure of gold nanodot swarm (AuNSw) and its application in photothermal therapy.

Here, we developed a novel PTT agent with a core-shell structure, composed of glycol chitosan (core) and small gold nanoparticles (shell), called a gold nanodot swarm (AuNSw) (Scheme 1). After hydrophobically-modified glycol chitosan formed particles (GCPs) in an aqueous solution, small gold nanoparticles (sGNPs) with a diameter of less than 20 nm assembled on the GCPs via the interaction between Au and amine functional groups of glycol chitosan. AuNSw generates heat upon NIR laser irradiation, as the absorption of gold aggregates shifts to the NIR region via the plasmon coupling effect [12,13]. This hyperthermal effect of AuNSw also induces ICD, causing immune responses against cancer. After the treatment the endogenous thiols, which are upregulated in cancer [14,15], replaced the bonds between GCPs and sGNPs and initiated dispersion of sGNPs from GCPs, as the interaction between Au and thiol groups was stronger than amine groups [16]. The disassembly of AuNSw prevented further damage under light irradiation.

## 2. Results

**Figure 1:**
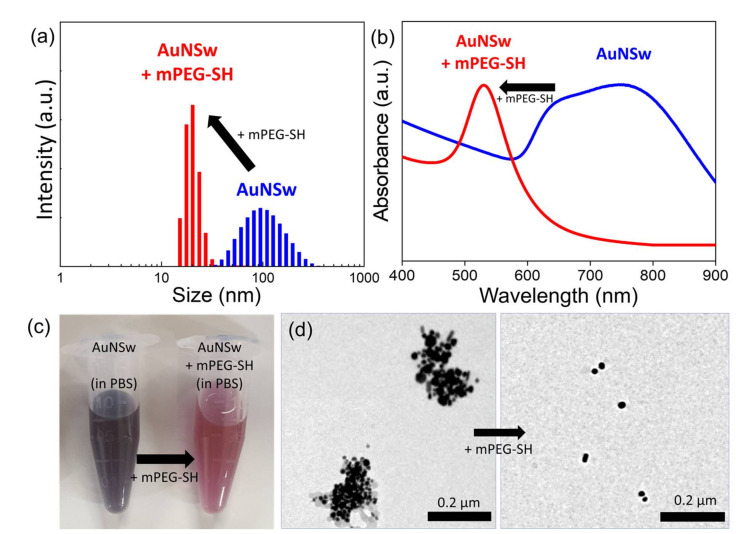
Characterization of AuNSw before and after the addition of mPEG-SH: (**a**) Size distribution of AuNSw measured by DLS changed from 156.50 ± 15.36 nm to 18.32 ± 11.25 nm after the addition of mPEG-SH; (**b**) The SPR peak of AuNSw shifted from 800 nm to 531 nm in PBS after the addition of mPEG-SH; (**c**) The color of AuNSw in PBS turned from dark purple to red after the addition of mPEG-SH; (**d**) TEM images illustrated AuNSw (left) without mPEG-SH and dispersed sGNPs after the addition of mPEG-SH (right).

We characterized the properties of AuNSw, which changed its size and optical properties in response to thiolated molecules. Measured by dynamic light scattering (DLS) in Figure 1a, the size of the AuNSw was 156.50 ± 15.36 nm in phosphate buffer saline (PBS). After exposure to the thiolated polyethylene glycol (mPEG-SH, MW = 6000) that substituted for endogenous reducing agent, the structure of AuNSw was disrupted, and dispersing sGNPs of 18.32 ± 11.25 nm. UV–vis spectrum demonstrated the optical property according to the states of AuNSw. The surface plasmon resonance (SPR) peak of AuNSw in PBS was at around 800 nm before the addition of mPEG-SH, while it shifted to 531 nm, the characteristic SPR peak of sGNPs, after the addition of mPEG-SH (Figure 1b). The change in the color of AuNSw colloid was also visible with the naked eye from dark purple to red due to the shifting of the SPR peak (Figure 1c). These changes originated from the different states of morphologies that were proven by transmission electron microscopic (TEM) images. The TEM images clearly showed the existence of the gold nanoparticles as black dots [17,18,19,20,21]. Figure 1d displayed the assembly of sGNPs in AuNSw before the addition of mPEG-SH (left) and dispersed sGNPs after the addition of mPEG-SH (right).

**Figure 2:**
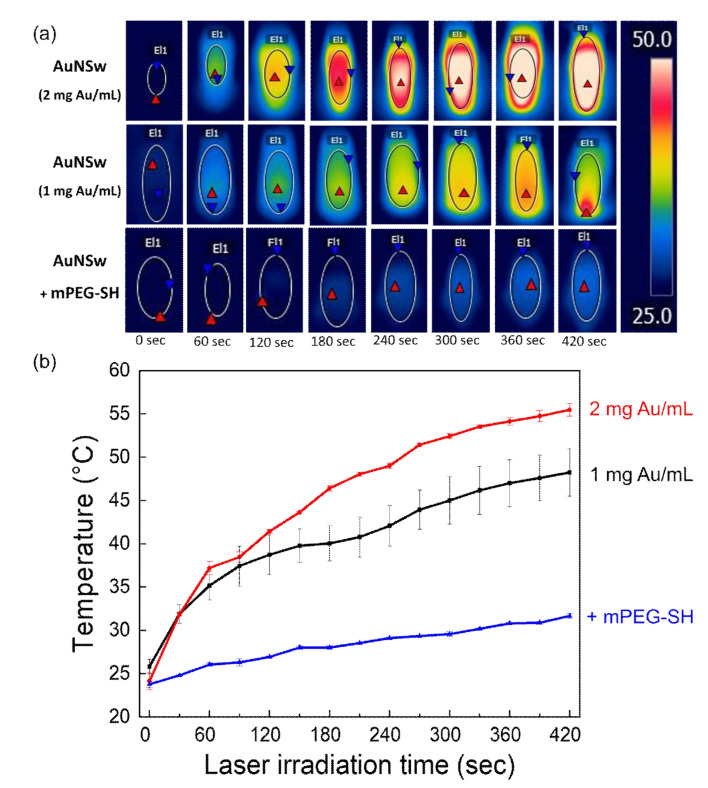
The photothermal effect of AuNSw upon CW laser irradiation (808 nm, 1 W/cm^2^): (**a**) Real-time thermal images displayed heat generation from AuNSw after CW laser irradiation, ac-cording to concentration (top: 2 mg Au/mL, middle: 1 mg Au/mL). If the particle structure was disrupted by mPEG-SH, AuNSw did not generate heat upon CW laser irradiation (bottom: 1 mg Au/mL after the addition of mPEG-SH); (**b**) Temperature changes were quantitatively measured (red: 2 mg Au/mL, black: 1 mg Au/mL, blue: 1 mg Au/mL after the addition of mPEG-SH).

We investigated the photothermal effect of AuNSw. Real-time thermographic camera and temperature measurement exhibited the temperature rise by AuNSw upon the irradiation of continuous-wave (CW) laser with the wavelength of 808 nm and power of 1 W/cm^2^ (Figure 2). The temperature increased to 55.5 °C within seven minutes of CW laser irradiation if the concentration of AuNSw was 2 mg Au/mL. If the concentration of AuNSw decreased, the rate of temperature changes also dropped, resulting in a saturated temperature at 48.2 °C. However, AuNSw was not able to generate heat upon CW laser irradiation when the particle structure was disturbed by mPEG-SH, and was unable to absorb the laser.

**Figure 3:**
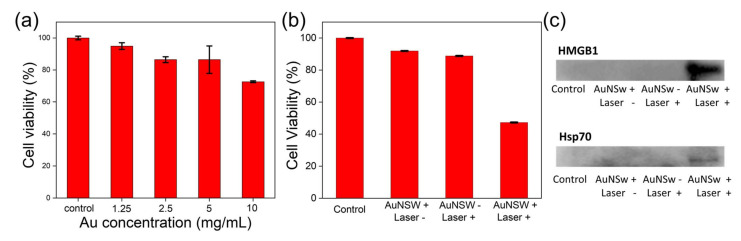
Cytotoxicity and therapeutic effect of AuNSw with CW laser irradiation: (**a**) Cytotoxicity of AuNSw among CT26 cancer cells after cellular uptake (12 h) with different AuNSw concentrations; (**b**) Cell viability showed the therapeutic effect of AuNSw (2.5 mg Au/mL) combined with CW laser irradiation (808 nm, 1 W/cm^2^, 10 min). Other groups did not exhibit toxic effects. Cellular uptake time was 12 h; (**c**) The expression of HMGB1 and Hsp70 was analyzed through western blot with samples obtained at 24 h after treatment.

We performed in vitro testing of the cytotoxicity and therapeutic effect of AuNSw with CT26 colon cancer cells. After the cells grew with various concentrations of AuNSw for 12 h, their viability was measured (Figure 3a). Cell viability testing revealed that AuNSw with 2.5 mg Au/mL had mild cytotoxicity (86.40 ± 1.79%) compared with the control group. However, if combined with CW laser irradiation, AuNSw showed an enhanced therapeutic effect on cancer cells (47.26 ± 2.54%) compared with other groups, such as cells with AuNSw alone (91.92 ± 0.53%) or with laser alone (88.83 ± 2.69%), as shown in Figure 3b. During cell death, CT26 cancer cells treated with both AuNSw and laser expressed biomarkers for ICD such as high mobility group box 1 (HMGB1) and 70 kilodalton heat shock proteins (Hsp70) (Figure 3c).

**Figure 4:**
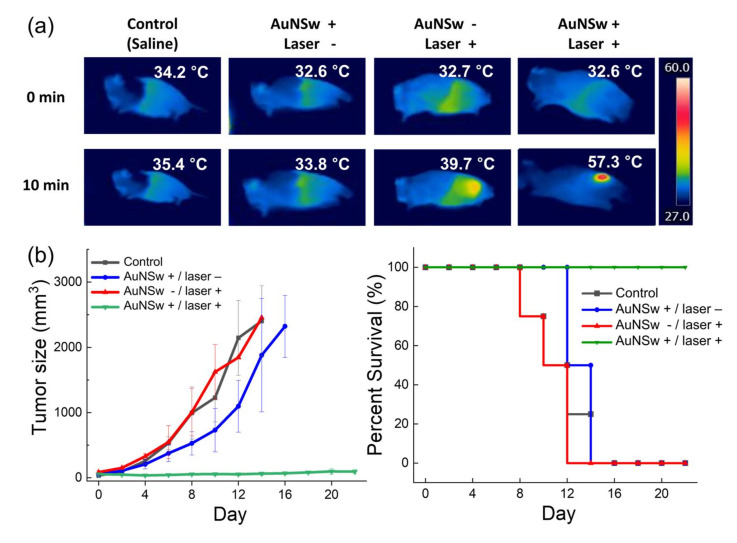
The photothermal therapeutic effect of AuNSw with CW laser irradiation: (**a**) Real-time in vivo thermal images of CT26-bearing mice with various treatments (Mice in the control group were treated with saline). The amount of intratumoral injection of AuNSw was 20 μL (2.5 mg Au/mL) and the CW laser irradiation time was 10 min (808 nm, 1 W/cm^2^); (**b**) Tumor growth curves (left) and survival rates (right) of CT26-bearing mice after various treatments (n = 4). Mice with tumor sizes over 2000 mm^3^ were counted as dead.

Real-time in vivo thermographic images revealed the photothermal effect of AuNSw in the body (Figure 4a). We divided CT26-bearing mice into four groups: control, laser-treated, AuNSw-treated, and laser- and AuNSw-treated groups. Each group was treated with intratumoral injection of AuNSw (20 μL, 2.5 mg Au/mL) and/or CW laser irradiation (10 min, 808 nm, 1 W/cm^2^) accordingly. The temperature in the tumor region increased to 57.3 °C if AuNSw and CW laser irradiation were used for simultaneously treatment. In contrast, it rose to only 39.7 °C if the animal models were treated with laser irradiation without AuNSw injection. The other two groups showed insignificant temperature increments.

We investigated the therapeutic effect against cancer after each group was treated differently. Tumor size did not increase for 22 days if the tumor was treated with both AuNSw and laser irradiation (Figure 4b, left). However, the tumor volume of the other three groups increased rapidly within 15 days until it reached 2000 mm^3^ (Figure S1, see Supplementary Materials). Data on survival rate demonstrated the same results (Figure 4b, right). The survival rate of the mice treated with both AuNSw and laser irradiation was 100% during the observation period. In contrast, mice from the other three groups failed to survive more than 15 days.

**Figure 5:**
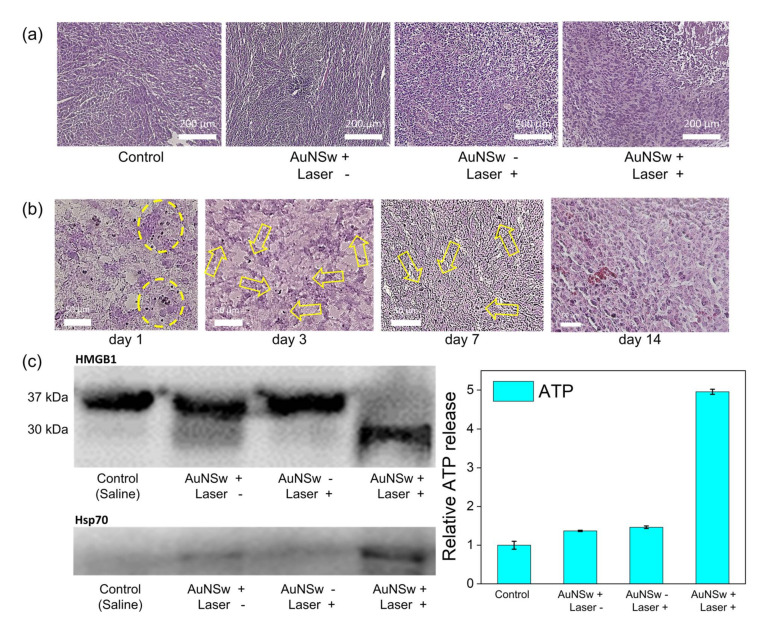
Histological and biochemical analysis of tumor tissues after the treatment: (**a**) Histological analysis was performed with tumor tissue samples, excised on 14 days after various treatments; (**b**) Histological images of tumors were obtained on 1, 3, 7, and 14 days after intratumoral injection of AuNSw. The silver staining emphasizes the location of gold nanoparticles (yellow circle or arrows); (**c**) Western blot analysis displayed the expression of HMGB1 and Hsp70 from tumor tissue after PTT (left). The level of ATP expression, measured with an ATP assay kit, increased after PTT (right).

After treatment, we harvested tumor tissues for histological and biochemical studies. Microscopic images of tumor tissues treated with AuNSw and laser indicated a damaged region (Figure 5a), while no tissue damage was found in the other three groups. Further histological analysis with a silver enhancer kit displayed the locations of gold nanoparticles in tumor tissues (Figure 5b). At 24 h after injection, assembled sGNPs remained inside the tissue (yellow circles in “day 1”). Then AuNSw began to lose its particle integrity, and sGNPs dispersed throughout the tissue after three days (yellow arrows in “day 3”). We observed fewer particles in the tissue after seven days (yellow arrows in “day 7”) and eventually no sGNPs after 14 days. Histological images of other organs did not show systemic toxicity during this period (Figure S2, see Supplementary Materials). Biochemical analysis of tumor tissues verified the ICD caused by PTT. The expression of HMGB1 and Hsp70 in tissues treated with AuNSw and laser irradiation was distinctively visible in the western blot analysis (Figure 5c). In addition, the expression of adenosine triphosphate (ATP), measured with an ATP assay kit, also significantly increased after the treatment.

## 3. Discussion

Spherical gold nanoparticles typically have their SPR peak near 530 nm in the UV-vis spectrum [21]. This optical absorption property is not preferable for PTT because light with a shorter wavelength has shallow penetration depth into the body [22]. As a therapeutic agent for PTT, gold nanoparticles should absorb light in the NIR region for efficient heat conversion in the body during PTT. The aggregated form of gold nanoparticles enables hyperthermia under NIR irradiation when plasmon coupling takes effect in the proximity of gold nanoparticles and shifts the optical absorption of gold nanoparticles to the NIR region [12,13]. Therefore, we designed AuNSw to form gold nanoparticle assemblies. The particle structure of AuNSw consisted of a GCP core with amine functional groups and superficial sGNPs via interactions between amine groups and sGNPs. These sGNPs assembled temporally due to their weak interactions with amine groups, and their SPR peak shifted to the NIR region due to the plasmon coupling effect [19]. We showed the morphology of AuNSw with assembled sGNPs and the corresponding SPR peak at the NIR region in Figure 1.

Moreover, AuNSw was capable of responding to the thiol-rich microenvironment of tumors and changing the particle structure. Because thiols on the gold surface form covalent bonding that is stronger than amine functional groups [16], thiolated molecules substituted glycol chitosan and disassembled the structure of AuNSw. If thiol groups of PEG intervened in the interactions between the amine groups and sGNPs, sGNPs emanated from GCPs and existed as a separate gold spheres. As a result, sGNPs recovered their original properties, e.g., the SPR peak at 530 nm and their size of approximately 20 nm. These properties closely relate to the appearance of colloid (Figure 1c) and to nanoparticle morphology (Figure 1d). In this status, sGNPs were not able to generate heat upon NIR laser irradiation, as their light absorption was too low. This reducing-environment-responsive property of AuNSw is beneficial to clinical applications in cancer treatment because endogenous thiols are upregulated in cancer [14,15]. After treatment, endogenous thiols would release sGNPs (< 20 nm), which would be advantageous for renal clearance [23]. We proved the thiol-responsive property of AuNSw with DLS, UV-vis spectrum, and TEM images.

The NIR absorption of AuNSw led to heat generation upon CW laser irradiation. The temperature increased 55.5 °C within 7 min and saturated afterward if the concentration of AuNSw was 2 mg Au/mL. Because PTT requires a temperature of more than 50 °C [24], we were able to apply AuNSw as an in vitro PTT agent. AuNSw did not show noticeable toxicity, either without CW laser irradiation or vice versa. Due to this low toxicity, AuNSw did not affect immune responses prior to PTT [25]. However, upon exposure to the laser, AuNSw produced enhanced toxicity among CT26 cancer cells. Cell viability after PTT barely reached 47%, as shown in Figure 3b, because the CW laser beam did not cover the whole area of each well in the well plate. These dying cells also released HMGB1 and Hsp70 as a result of ICD. We expected that ICD would induce additional anti-cancer immune responses in the tumor microenvironment.

The concentration and particle structure of AuNSw were critical factors for the control of temperature ascent during PTT. If the concentration halved, then the temperature increased until it reached 48.2 °C. In addition, if thiolated molecules disrupted the structure of AnNSw, then sGNPs dispersed from AuNSw, leading to the disappearance of the plasmon coupling effect. As a result, they lost their NIR absorption property as well as the photothermal effect upon 808-nm laser irradiation. Therefore, reducing-environment-responsive AuNSw has two advantages: (1) Disassembled AuNSw in the tumor microenvironment did not generate heat, preventing additional damage upon exposure to light after PTT; (2) Residual sGNPs released from AuNSw by endogenous thiols undergo renal clearance after PTT.

These in vitro results became the basis of in vivo testing of AuNSw for PTT. We observed the hyperthermic effect of AuNSw with CW laser irradiation through a thermographic camera. AuNSw in the same concentration generated heat upon NIR laser irradiation in the body, because NIR light has a deeper penetration depth in the body. The high temperatures resulted in therapeutic effects on cancer. Tumor growth and survival rate curves indicated that the treatment with both AuNSw and laser irradiation reduced tumor volume among animal models for 22 days. In contrast, other treatment methods in the control groups failed to prevent tumor growth.

Histological and biochemical analyses also illustrated the therapeutic effect of AuNSw with CW laser irradiation. Microscopic images of tumor tissue treated with AuNSw and laser revealed tissue damage caused by the hyperthermal effect. This cell damage was also accompanied by ICD, which was proven by western blot for HMGB1 and Hsp70. HMGB1 and ATP are DAMPs of ICD that accelerate phagocytosis of dendritic cells and promote tumor antigen presentation to T cells [26]. This ICD by PTT with AuNSw may further expedite the inhibition of tumor growth along with the hyperthermal effect. Histological images with silver staining revealed the behavior of AuNSw in the tumor microenvironment. In the early stage of intratumoral injection of AuNSw, we observed a group of sGNPs in the tissue. Afterward, sGNPs dispersed throughout the tissue, and fewer sGNPs were located in the histological images. We expected that the reducing-environment responsive properties of AuNSw would be valuable for clinical applications because of the clearance of sGNPs due to their small size.

To conclude, the characterization of AuNSw with DLS, UV–vis spectrum, and TEM images demonstrated the successful synthesis of AuNSw. AuNSw absorbed NIR laser and caused hyperthermia as well as ICD. The innovative feature of AuNSw was in the shifting of optical absorption in response to reducing agents that are abundant in the tumor microenvironment. Once the endogenous thiols dispersed sGNPs from AuNSw the CW laser irradiation did not cause a temperature increase, due to the discrepancy of the SPR peak of AuNSw and the wavelength of the NIR laser. The loss of the photothermal effect of AuNSw prevents unnecessary damage after PTT. Moreover, we assume that the small size of sGNPs was advantageous for clearance from the body after PTT [27]. Therefore, thiol-responsive AuNSw had valuable features for the clinical applications of PTT.

## 4. Materials and Methods

### 4.1. Materials

Polyethylene glycol methylether thiol (mPEG-SH, MW = 6000), HAuCl_4_·3H_2_O (99.9%,), 5β-cholanic acid (99%), N-(3-dimethylaminopropyl)-N-ethylcarbodiimide hydrochloride (EDC, commercial grade) N-hydroxysuccinimide (NHS, 98%), Sodium borohydride (NaBH_4_, 99%), and silver enhancer kit (SE100-1KT) were purchased from Sigma-Aldrich (St. Louis, MO, USA). Glycol chitosan (MW = 205.22) and Cell Counting Kit-8 (CCK-8) were obtained from MP Biomedicals (Santa Ana, CA, USA) and Dojindo Laboratory (Kumamoto, Japan), respectively. Materials for cell culture, such as RPMI-1640, fetal bovine serum (FBS), and antibiotic-antimycotic (AA) were acquired from GenDEPOT (Katy, TX, USA). Water used in the experiment was distilled and all chemicals were used without any purification.

### 4.2. Synthesis of Hydrophobically-Modified Glycol Chitosan Particles (GCPs)

GCPs were synthesized through the conjugation of glycol chitosan with 5β-cholanic acid, as described previously [28]. Briefly, aqueous solutions of glycol chitosan (125 mL, 4 mg/mL), 5β-cholanic acid (150 mg), EDC (120 mg), and NHS (72 mg) were mixed under stirring for 24 h. Then, the reacted solution was dialysed for three days with a dialysis membrane (MWCO: 12,000−14,000) in methanol/water solvent mixture (1:4, *v*/*v*). After dialysis, the solution was filtrated with a 0.8 μm syringe filter and freeze-dried at −85 °C, 57 mTorr for three days. GCP colloid was prepared by dissolving 2 mg of hydrophobically-modified glycol chitosan in 1 mL of water under sonication for 10 min.

### 4.3. Synthesis and Characterization of Gold Nanodot Swarm (AuNSw)

An aqueous solution of HAuCl_4_·3H_2_O (1 mL, 4 mg/mL) and NaBH_4_ (0.2 mL, 1.9 mg/mL) was prepared in ice-cold water. After mixing GCP colloid with HAuCl_4_·3H_2_O solution, NaBH_4_ solution was quickly added to the mixed solution, which was kept under stirring at room temperature for 24 h. The AuNSw was then washed twice through centrifugation at 10,000 rpm for 25 min in Smart R17 (Hanil Science industrial Co., Ltd., Seoul, Korea) and re-dispersed for 5 min with sonicator, Ultrasonic Cleaner Set (DAIHAN Scientific, Busan, Korea). The size distribution and optical absorption properties of AuNSw were measured in Zetasizer Nano ZS (Malvern Instruments, Malvern, UK) and UV-Vis Spectrophotometer (Agilent Technologies, CA, USA), respectively. The morphology of AuNSw was captured in Tecnai TEM (FEI, Hillsboro, OR, USA) operating at 200 kV. TEM samples were prepared on carbon-coated, 400-mesh copper grids by dropping 10 μL of AuNSw, followed by dehydration in a vacuum oven for 12 h. The same characterization methods were applied to AuNSw after the addition of mPEG-SH (1 mL, 2 mg/mL). The photothermal effect of AuNSw was evaluated with a real-time thermographic camera, E5-XT (FLIR, Wilsonville, OR, USA) and thermocouple (OMEGA, Norwalk, CT, USA) after irradiation with a CW laser (Changchun New Industries Optoelectronics Tech. Co., Ltd., Changchun, China) with the wavelength of 808 nm and the power of 1 W/cm^2^.

### 4.4. In Vitro Tests of Cytotoxicity and Therapeutic Effect of AuNSw

Cytotoxicity and therapeutic effect of AuNSw were tested with CT26 cancer cells. Cells were cultured in RPMI-1640 medium containing FBS (10%, *v*/*v*) and AA (1%, *v*/*v*) in 96-well plates with a density of 10^4^ cells/well at 37 °C, 5% CO_2_. Then, cells were treated with different concentrations of AuNSw (1.25, 2.5, 5, and 10 mg Au/mL) for 12 h and washed with PBS buffer before the measurement of cell viability using CCK-8.

The therapeutic effect of AuNSw was evaluated through cell viability measurement using CCK-8. The concentration of AuNSw was fixed to 2.5 mg Au/mL for cellular uptake. Cells were washed three times with PBS before CW laser irradiation for 10 min. The therapeutic effect was compared with other groups, such as cells with only AuNSw uptake, or with laser irradiation alone.

### 4.5. In Vivo Study

Animal experiments were performed as approved by the Institutional Animal Care and Use Committee of Korea Institute of Science and Technology under protocol KIST-2020–114 (date of approval: November 11, 2019−November 01, 2021). Six-week-old male Balb/C mice were purchased from NaraBio, Inc. (Seoul, Korea) and tumor xenograft models were produced with 100 μL of CT26 cell suspension (106 cells). After seven days, animal models were randomly divided into four groups: control, laser-treated, AuNSw-treated, and laser- and AuNSw-treated groups. Each group was treated with intratumoral injection of AuNSw (20 μL, 2.5 mg Au/mL) and/or CW laser irradiation (10 min, 808 nm, 1 W/cm^2^), respectively. Thermal images of mice in the four groups were acquired before and after laser irradiation. Tumor sizes and survival rates of the four groups (n = 4) were measured after treatment. The measurements were continued for 22 days or until the tumor size reached approx. 2000 mm^3^. The tumor volume was calculated as follows:

Tumor volume = length × width^2^ × 0.53 (1)

where length was the longest axis of the tumor and width was the diameter of the tumor, perpendicular to the length.

### 4.6. Histological and Biochemical Analyses

For histological analysis, tumor tissues were collected and fixed in PBS containing 4% paraformaldehyde. After embedding in paraffin, tissue samples were sectioned at a thickness of 10 μm. Then, samples were stained with hematoxylin and eosin. Additionally, silver staining was used for the visualization of gold nanoparticles in the tumor tissues. The samples were observed on DP71 microscope (OLYMPUS, Tokyo, Japan).

The release of HMGB1 and Hsp70 was analyzed with CT26 cells incubated with AuNSw (2.5 mg Au/mL) for 12 h in 6-well plates (cell density: 1 × 10^6^ cell/well) and irradiated by CW laser for 10 min. Then, after another 24 h, supernatant cell culture media was collected and qualitatively analyzed with western blot. For in vivo analysis, tumor tissues were harvested on day five after treatment. Tissue samples were mixed with RIPA buffer and centrifuged. The supernatant of tumor samples was analyzed with western blot. ENLITEN^®^ ATP assay (Promega, Madison, WI, USA) was purchased for the analysis of ATP. A mixture of 200 μL of supernatant samples and rL/L reagent was mixed (1:1, *v*/*v*) in a 96-well plate and the fluorescence was measured in IVIS Lumina (Caliper LifeSciences, Waltham, MA, USA). The exposure wavelength and time were 300 nm and 5 min, respectively.

## Data Availability

Not applicable.

## Supplementary Materials

The following are available online, Figure S1: Representative photographs of CT26-bearing mice after different treatments, Figure S2: Histology of other organs on 14 days after injection of AuNSw.

## Sample Availability

Samples of the compound are available from the authors.
