# Supplementary material for: Thiol-Responsive Gold Nanodot Swarm with Glycol Chitosan for Photothermal Cancer Therapy
Source: Molecules. 2021 Oct 2;26(19):5980. doi: 10.3390/molecules26195980 (PMC8512322; doi:10.3390/molecules26195980)
Supplement: Supplementary file 1 [file molecules-26-05980-s001.zip › molecules-1375255-supplementary.pdf]

Supplementary Materials

Thiol-responsive gold nanodot swarm with glycol chitosan for photothermal cancer therapy

SeongHoon Jo [1,2,†], In-Cheol Sun [1,†], Wan Su Yun [3], Jinseong Kim [3], Dong-Kwon Lim [3], Cheol-Hee Ahn [2,*], and Kwangmeyung Kim [1,3,*]

[1] Center for Theragnosis, Biomedical Research Institute, Korea Institute of Science and Technology, 5, Hwarang-ro, Seongbuk-gu, Seoul 02792, Korea; jsh@kist.re.kr (S.J.); pfesun@kist.re.kr (I.-C.S.)

[2] Research Institute of Advanced Materials (RIAM), Department of Materials Science and Engineering, Seoul National University, 1 Gwanak-ro, Gwanak-gu, Seoul 08826, Korea

[3] KU-KIST Graduate School of Converging Science and Technology, Korea University, 145 Anam-ro, Seongbuk-gu, Seoul 02841, Korea; ip9801@kist.re.kr (W.S.Y.); 218843@kist.re.kr (J.K.); dklim@korea.ac.kr (D.-K.L.)

* Correspondence: chahn@snu.ac.kr (C.-H.A.); kim@kist.re.kr (K.K.)

† These authors contributed equally to this work.

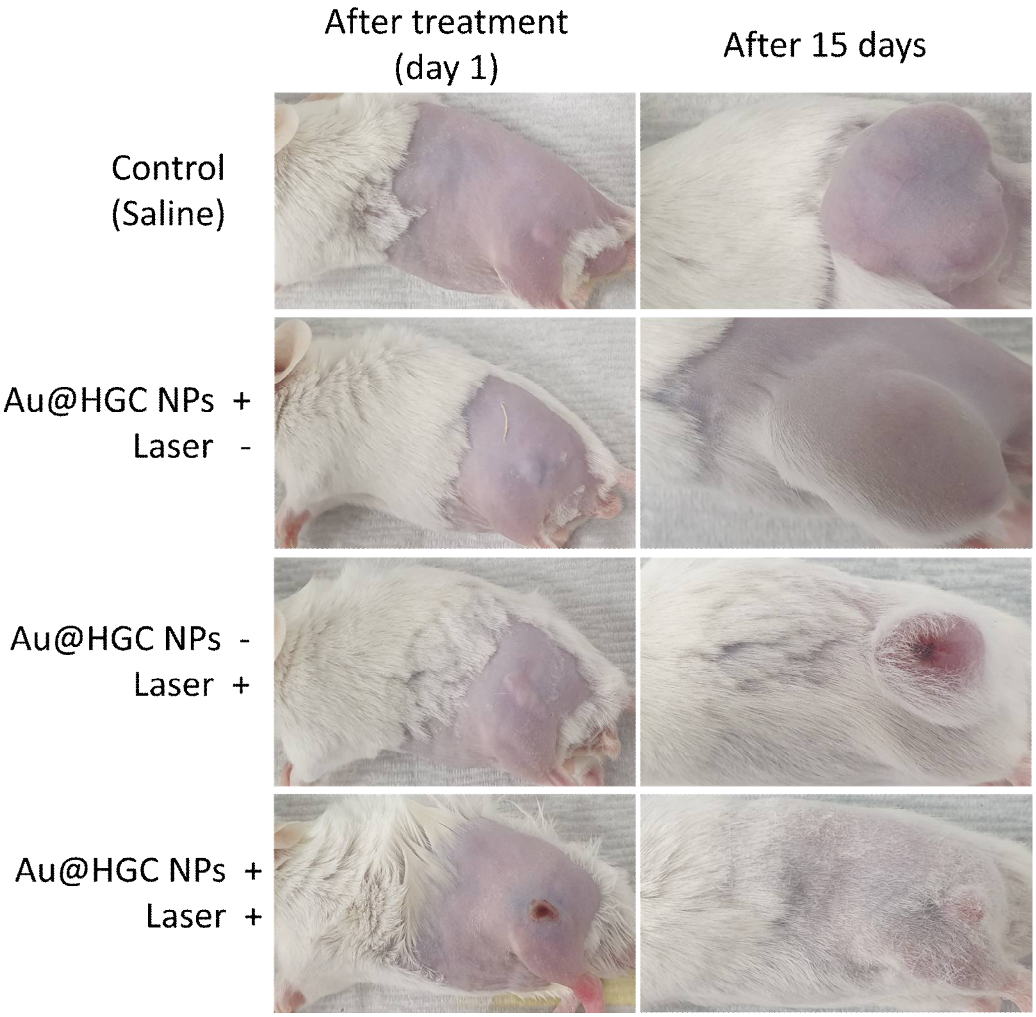

Figure S1. Representative Photographs of CT26-bearing mice after different treatments.

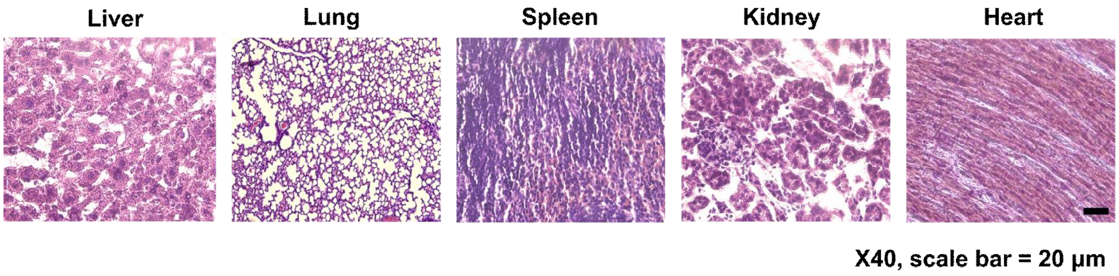

Figure S2. Histology of other organs on 14 days after injection of AuNSw.
